# The Effects of Presleep Slow Breathing and Music Listening on Polysomnographic Sleep Measures – a pilot trial

**DOI:** 10.1038/s41598-020-64218-7

**Published:** 2020-05-04

**Authors:** Liisa Kuula, Risto Halonen, Kristiina Kajanto, Jari Lipsanen, Tommi Makkonen, Miina Peltonen, Anu-Katriina Pesonen

**Affiliations:** 1 SleepWell Research Program, Faculty of Medicine, University of Helsinki, Helsinki, Finland; 2 Department of Psychology and Logopedics, Faculty of Medicine, University of Helsinki, Helsinki, Finland; 3 Department of oral and maxillofacial diseases, Faculty of Medicine, University of Helsinki, Helsinki, Finland

**Keywords:** Circadian rhythms and sleep, Human behaviour, Quality of life

## Abstract

Knowledge on efficient ways to reduce presleep arousal and, therefore, improve sleep, is scanty. We explored the effects of presleep slow breathing and music listening conditions on sleep quality and EEG power spectral density in young adults in a randomized, controlled trial with a crossover design. Participants’ (N = 20, 50% females) sleep was measured on two consecutive nights with polysomnography (40 nights), the other night serving as the control condition. The intervention condition was either a 30-minute slow breathing exercise or music listening (music by Max Richter: Sleep). The intervention and control conditions were placed in a random order. We measured heart rate variability prior to, during and after the intervention condition, and found that both interventions increased immediate heart rate variability. Music listening resulted in decreased N2 sleep, increased frontal beta1 power spectral density, and a trend towards increased N3 sleep was detected. In the slow breathing condition higher central delta power during N3 was observed. While some indices pointed to improved sleep quality in both intervention groups, neither condition had robust effects on sleep quality. These explorative findings warrant further replication in different populations.

## Introduction

Sleep problems are common in today’s society. In a multi-national cross-sectional survey, as many as 37% of adults reported sleep complaints, while 35% reported having difficulty initiating and maintaining sleep or non-restorative sleep at least three days per week^1^. Given the high prevalence of sleep problems, there is an urgent need for sleep-promoting interventions that can be applied in a self-directed way.

Presleep arousal refers to the mental and physiological activation that individuals experience when trying to fall asleep. Presleep arousal may lead to disrupted autonomic nervous system (ANS) activity during sleep^2,3^, and has been shown to mediate the association between higher stress and poorer sleep^4–6^. Slow breathing (i.e., breathing at a frequency close to 0.1 Hz) may be a simple way to reduce presleep arousal since it enhance vagal activity^7,8^ and increase feelings of relaxation^7,9^.

Indeed, recent research has demonstrated that slow breathing performed before or at bedtime can improve sleep as measured by polysomnography (PSG). Relative to a baseline measurement, a 20-minute slow breathing session (performed before bedtime at a frequency of 0.1 Hz) was shown to reduce wake after sleep onset (WASO), lower the percentage of N2 sleep, and reduce sleep onset latency (SOL), as well as improve subjective sleep quality in 14 self-reported insomniacs^10^. No differences were found between the intervention night and the baseline measurement in a control group consisting of 14 good sleepers. A randomized controlled trial (RCT) (N = 10) found that a single 20-minute biofeedback-assisted slow breathing session before bedtime resulted in an improved score on a sleep disturbance scale (derived from sleep efficiency, rapid eye movement (REM) latency, minutes of stage N1 sleep, and WASO) when compared to a no-treatment control condition^11^.

Furthermore, a randomized controlled crossover trial^12^ investigated the effects of slow breathing (performed at bedtime at a frequency of 0.1 Hz) on PSG-measured sleep of 16 women with insomnia symptoms. The authors report that following the slow breathing intervention, participants had a reduced number of awakenings and sleep stage transitions as compared to a control night, but no changes in other PSG parameters were detected. However, their study included audio-visual stimulation to guide participants to reach the desired breathing rate, making it difficult to differentiate the effects of slow breathing from those of the visual and auditory stimuli.

It should be noted that previous studies have not examined whether the observed improvements in sleep are due to the chosen breathing technique or a general relaxation effect. To address this issue, the current study examined the impact of slow-breathing on PSG-measured sleep in parallel to that of relaxing music listening, a popular self-help strategy to enhance sleep onset^13^.

Studies of the effects of music listening before or at bedtime on PSG-measured sleep have yielded very mixed results, and most of them have focused on insomnia, which may have heterogeneous etiology and symptomatology^14^. An RCT of 50 self-reported insomniacs found that listening to music before bedtime resulted in shorter stage N2 sleep and longer REM sleep when compared to a control group not exposed to music^15^. However, another study (N = 57) did not find a decrease in the insomnia severity index in a music listening paradigm that lasted for three weeks^16^.

In non-clinical populations, the findings have been equally sporadic. Lazic and Ogilvie^17^ studied 10 female participants with normal sleep using a within-subjects design and found no differences in PSG variables between music, general auditory stimulation, or control (no auditory stimulation) conditions. There were, however, minor differences in delta powers during sleep onset and the first slow wave sleep SWS period between these conditions. In a randomized controlled crossover trial of 24 individuals with no sleep disturbances, listening to sedative music before bedtime led to prolonged duration of slow wave sleep (but only in individuals with long sleep latency) and to decreased duration of stage N2 sleep^18^. Finally, Cordi *et al*. found that music listening, in contrast to text reading, before napping improved subjective sleep quality and decreased N1 sleep in healthy subjects (N = 24), but in highly suggestible individuals it also increased of SWS^19^.

Sleep complaints are highly common even in non-clinical populations. More information is needed to understand what individuals with prolonged sleep latency or discontinuous sleep can themselves do to improve their sleep quality. To address the paucity of research in this area, we set out a randomized controlled crossover trial to examine the effects of presleep slow breathing and music listening on PSG-measured sleep structure and N3, non-REM (NREM), and REM sleep power spectra. While the previous evidence is scarce, we set an initial hypothesis that music and slow breathing would both improve sleep, by decreasing SOL and increasing sleep continuity and the amount of SWS. There is currently very little evidence on the effect of presleep relaxation activities on EEG power density, thus, our approach is then exploratory. We also explored how participants responded to the interventions as measured by the ANS reactions, and how this response was reflected on sleep.

## Methods

### Study design

**Figure 1:**
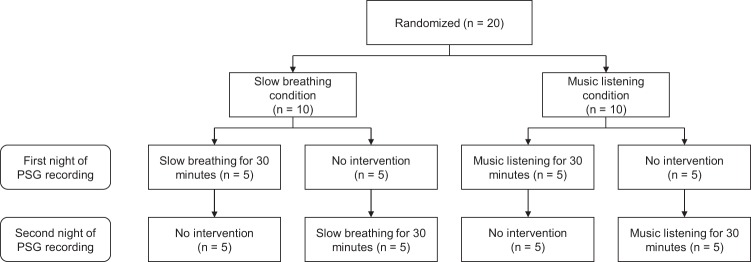
Study Design.

We set up a two-arm randomized controlled trial with a crossover design to evaluate the effectiveness of presleep relaxation in improving sleep quality. Participants were randomly assigned using random numbers to either having a slow breathing exercise before bedtime (our primary intervention), or to listening to relaxing music before going to bed (our secondary, explorative intervention). Participants were measured at two consecutive nights, and the order of the nights for the experimental or no-treatment conditions was also randomized. The evening without a breathing exercise or music listening was used as a control condition and defined as a usual evening at home. The study design is illustrated in Fig. 1. This study is registered with ClinicalTrials.gov, number NCT03657901 (05/09/2018).

Power calculations were done by simulation, using simr-package^20^ in R-software version 3.6.2 (R Core Team, 2019, Austria) we concluded that using the available sample size of 20 we could detect large effect sized as statistically significant with 80% power. Also observed power was calculated as a benchmark for future research and as expected ranged from 50–80% for statistically significant results and from 5–30% for insignificant.

### Participants

Inclusion criteria were age between 20 and 45 years and a relatively stable sleep schedule (e.g., no shift work or jet lag). Exclusion criteria were any diagnosed sleep disorder, the use of any medication that could affect sleep, acute sickness (e.g., the flu), and gold allergy (as electrodes used for the PSG recording were gold-plated). The inclusion and exclusion criteria were controlled based on self-reports. The voluntary sample included 20 individuals (mean age 24.5 years, standard deviation (SD) 3.5, range 20–37 years; 50% females) whose sleep was measured for two nights. Each participant received a compensation of 100 euros and structured feedback on their sleep stages. Written informed consent was obtained from all participants. The study was approved by the Ethical Committee of the Helsinki University Central Hospital. All procedures followed were in accordance with the Helsinki Declaration and its later amendments.

### Intervention conditions

In the slow breathing exercise, participants took five breaths per minute for 30 minutes (at a frequency of 0.08 Hz), starting approximately one hour prior to the expected bedtime. The exercise was conducted with a smartphone app “The Breathing App” (by Sergey Varichev, freely available in Google Play and App Store). The app had been programmed to work with the desired breath ratio and for the predefined length of time. Participants were instructed to choose the screen view with sound cues for the selected breathing pace.

In the music listening condition, participants listened the first two tracks and the first part of the third track on Max Richter’s album “Sleep”^21^ (duration 31 minutes). The selected 30 minutes are ambient, calm, and slow. The music was composed for piano and string instruments, as well as the organ, soprano vocals, synthesizers and electronics. This piece of work was originally intended to work as a sleep aid. Participants used headphones (JBL, Harman International Industries, Inc.) and were able to adjust the volume to their comfort level. Participants were allowed to close their eyes during both interventions, but they were instructed to stay in a sitting position so as not to fall asleep. Despite this, one subject fell asleep during music listening. One participant reported back pain and did the breathing exercise lying down.

### Variables and measures

#### *Subjective sleep quality*

The Finnish version of the Pittsburgh Sleep Quality Index (PSQI)^22^ was used to assess differences in sleep problems between the participants in slow breathing and music listening conditions. The PSQI is a self-report questionnaire consisting of 19 items that can be grouped into seven component scores. The global score ranges from 0 to 21, with lower scores indicating better sleep quality. A global score of six or greater indicates poor sleep quality. The PSQI has displayed strong reliability and validity^22,23^. In addition, subjective sleep quality following each night was measured using one question in the morning: “How did you sleep?” with response options ranging from 1 to 5 (1 = poorly, 2 = quite poorly, 3 = not good but not bad, 4 = quite well, 5 = well).

*Polysomnography*. PSG was measured at participants’ homes for two consecutive nights. 15 nights were measured with SOMNO HD^TM^(SOMNOmedics GmbH, Randersacker, Germany) device, and the remaining 25 were measured with SOMNOscreen^TM^plus (SOMNOmedics GmbH, Randersacker, Germany) device. Six electroencephalography (EEG) channels at F3, F4, C3, C4, O3, O4, and contralateral mastoids (A1 and A2) were recorded according to the international 10–20 system with a sampling rate of 256 Hz. In addition, two electro-oculogram channels and electromyogram on the chin were recorded. The ground electrode was placed on the forehead and the recording reference electrode over Cz. Sleep was scored manually in 30-second epochs with DOMINO software (version 2.9.0; SOMNOmedics GmbH, Randersacker, Germany) according to the American Academy of Sleep Medicine criteria^24^. Primary outcomes were SOL, WASO, and percentage of Total sleep time (TST) spent in N1, N2, N3, and REM sleep but we also report TST, REM sleep latency, and REM density.

### Power spectral density analysis

Signal processing was done using Matlab R2018a (The Mathworks Inc., USA) and functions of EEGlab 14.1.2b^25^. EEG signals were digitally filtered (passband 0.5–35 Hz; Hamming windowed sinc zero-phase FIR filter, cut-off (−6 dB) 0.25 Hz and 39.3 Hz respectively) and re-referenced to the average of mastoids. Pre-scored epochs having contact impedance equal to or lower than 20 kOhm in the target electrode and both mastoids were included in the power spectral density (PSD) analysis. We calculated PSD using the “spectopo” function of EEGlab (1024 samples and overlap of 50%). The signals were shifted positive by adding the absolute value of the global (all subjects, channels, and sleep stages) minimum to each spectrum. Mean values were then calculated for five frequency bands (0.5–3 Hz, 4–7 Hz, 8–12.5 Hz, 16–24 Hz, and 25–35 Hz).

The mean values of central (C3, C4) and frontal (F3, F4) PSD values were calculated separately for REM sleep, NREM sleep (N2 and N3), and stage N3 sleep over the entire night and during the first sleep cycle. Datasets having equal to or less than 10 valid 30-second epochs were excluded from the analyses. Due to high impedance levels in the mastoid electrodes, we excluded one night in the slow breathing condition and one night in the music listening condition from the EEG power spectral density analyses. No control nights were excluded. Thus, the number of nights for the power spectral density analyses was 9 in both conditions.

### Autonomous nervous system activity

The activity of the autonomous nervous system (ANS) was indirectly measured through heart rate (HR) and heart rate variability (HRV), with two non-invasive electrocardiography (ECG) electrodes placed on the chest (i.e., on the right side of the body right under the collarbone and on the left side of the body on the rib cage). ECG signals were collected at a rate of 1000 Hz using the Firstbeat Bodyguard 2 (Firstbeat Technologies, Jyväskylä, Finland) and analyzed with the FirstBeat Cloud Service. Resting HRV was measured for 30 minutes prior to intervention, during the 30-minute intervention and 30 minutes after. We used the Root Mean Square of Successive Differences in R-R intervals (RMSSD) as the HRV index, and to quantify the degree of sympathovagal balance between sympathetic and parasympathetic activity, we used the mean ratio between the low frequency (0.04–0.15 Hz) and high frequency (0.15–0.4 Hz) heart rate variability power (LF/HF) during the sessions (LF/HF ratio). We calculated an index of ANS reactivity to intervention ((increase of HRV or LF/HF ratio from baseline to intervention/baseline HRV or LF/HF ratio))*100).

### Procedure

A research assistant visited participants at their homes at two consecutive nights. Participants were asked not to consume alcohol or caffeine after 4 pm on the measurement nights. The evening visit started between 6 and 10 pm, depending on the participant’s typical sleeping schedule. All participants were free to select their bedtime according to their own circadian rhythm. The research assistant attached the electrodes to the participant and started the recording.

Before the research assistant left, participants were given instructions for the night. If they had no intervention that evening, they were instructed to spend the evening as usual, but refrain from vigorous activities. If they were assigned to do the breathing exercise or music listening that evening, a smartphone containing The Breathing App and music was provided to participants, and they were taught how to use their assigned app. Participants were asked to start the intervention approximately an hour before their estimated bedtime, and to use the time left after finishing the intervention for their evening routines (e.g., for brushing teeth, or eating an evening snack). The schedule was planned such that all participants would be equally exposed to the intervention rather than fall asleep during the exercises. Lastly, subjects were asked to keep their phones and any other electrical devices with transmitters at least two meters away from the bed so that they would not interfere with the PSG recording. Participants were instructed to sleep normally, and the visit for the following morning was scheduled according to the participant’s expected wake-up time. The research assistant arrived the following morning approximately 0 to 30 minutes after wake-up time and stopped the PSG recording.

### Statistical analyses

Differences in background variables between the individuals participating in the two conditions were studied using a Student’s t test for normally distributed continuous data (Body Mass Index, BMI; weight (in kilograms) divided by height (in meters) squared), a Mann-Whitney U test for not normally distributed continuous data (PSQI score and age), and a Fisher’s exact test for categorical data (sex and the number of subjects with poor self-reported sleep quality). Data for SOL, REM latency, WASO, and the percentage of N1 sleep were positively skewed, and, therefore, log10 transformed to achieve normal distribution.

We used repeated measures analysis of variance to study the effects of the two conditions on ANS activity. We used a linear mixed-effects model to compare differences in basic sleep parameters and power spectral density between the intervention and control conditions. The estimation method was restricted maximum likelihood. A random intercept model was used in all analyses. As a post-hoc analysis, we also examined how the ANS reactivity to intervention correlated with the PSG measures. All data analyses were carried out using SPSS software (version 25.0, IBM Corp., Armonk, NY, USA).

## Results

### Initial analyses across all conditions

Table 1 shows participant characteristics for the total sample and by intervention condition. The participants in the intervention conditions did not differ significantly from each other in age, gender composition, body mass index, and the PSQI score. Subjective sleep quality did not differ between the two measured nights (P = 0.8), or between the two different intervention conditions (M _slow breathing_ = 4.1 vs. M _music listening_ = 3.8; P = 0.39).

**Table 1:** Characteristics for the Total Sample and for Participants Randomized to Slow Breathing and Music Listening Conditions.

Characteristic	Total (n = 20)	Slow breathing (n = 10)	Music listening (n = 10)	p
	Mean (SD) or n (%)	Mean (SD) or n (%)	Mean (SD) or n (%)	
Age (years)	24.50 (3.50)	25.90 (4.15)	23.10 (2.08)	0.075
Sex (female)	10 (50.0%)	4 (40.0%)	6 (60.0%)	0.66
BMI (kg/m^2^)	23.64 (3.10)	23.90 (2.65)	23.38 (3.62)	0.72
PSQI score	5.40 (2.35)	6.10 (2.51)	4.70 (2.06)	0.075
Poor sleep quality (PSQI score > 5)	5 (25.0%)	4 (40.0%)	1 (10.0%)	0.30

Abbreviations: BMI, body mass index; PSQI score, Pittsburgh Sleep Quality Index global score.

**Figure 2:**
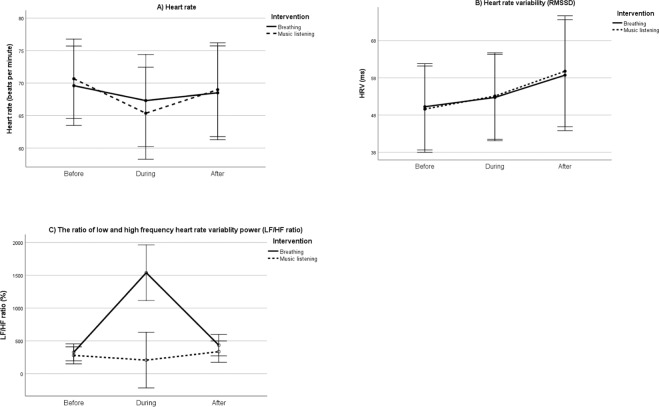
Heart rate indices in slow breathing and music listening situations. Y-axis shows the averaged values during the 30-minutes’ period prior to intervention, during the 30-minutes intervention and during the 30-minutes’ period after the intervention.

Figure 2 shows the effects of the slow breathing and music listening on ANS activity. The tests of within-subjects contrast were significant for all ANS parameters (HR: F_Quadratic_ = 9.99, P = 0.005; HRV: F_linear_ = 8.88, P = 0.008; LH/HF ratio: F_Quadratic_ = 19.2, P < 0.001), and ‘time*intervention’ was significant for LF/HF ratio (F = 27.2, P < 0.001; P > 0.18 for HR and HRV).

### Basic Sleep Parameters from PSG

**Figure 3:**
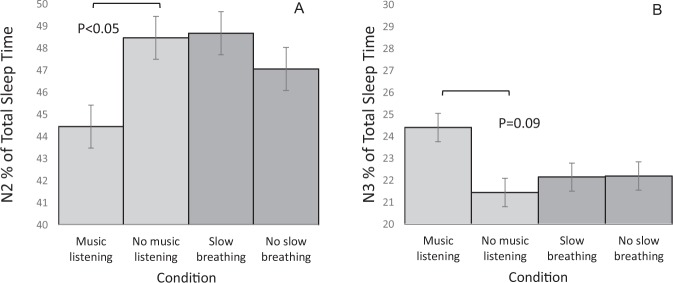
Percentages of N2 and N3 sleep from total sleep time according to presleep conditions.

The effects of the interventions on basic sleep parameters are shown in Table 2. There was a significant main effect of music listening on the percentage of stage N2(F = 4.325, P_condition_ = 0.049) and a trend regarding the percentage of N3 (F = 3.162, P _condition_ = 0.091) sleep. Music listening, compared to the control condition, was associated with a lower percentage of stage N2 sleep (estimated percentages 44.8% and 48.3%) and with a higher percentage of stage N3 sleep (estimated percentages 24.5% and 22.0%) (Fig. 3). There were no main effects for the slow breathing condition on the basic sleep parameters.

**Table 2:** Results of the Mixed-Effects Model Analyses for PSG Variables.

	Slow breathing (n = 10)	Music listening (n = 10)	Control (n = 20)	Intervention				
				Music listening			Slow breathing	
	Mean (SD)	Mean (SD)	Mean (SD)	F	p	F		p
TST (min)	448.19 (42.67)	435.52 (44.38)	454.28 (54.91)	1.16	0.29	0.30		0.59
SOL (min)^a^	12.56 (8.81)	20.59 (15.80)	16.52 (12.61)	0.78	0.39	0.74		0.40
REM latency (min)^a^	82.85 (25.63)	81.85 (45.74)	77.03 (20.25)	0.03	0.86	0.31		0.59
WASO (min)^a^	25.85 (25.35)	26.20 (37.06)	20.83 (18.29)	0.13	0.72	0.96		0.34
Stage N1 (%)^a^	5.14 (2.23)	4.14 (2.80)	4.71 (2.97)	1.65	0.21	0.85		0.37
Stage N2 (%)	48.68 (5.52)	44.45 (4.51)	48.36 (6.86)	4.33	0.049	0.01		0.94
Stage N3 (%)	22.15 (5.91)	24.41 (8.58)	21.09 (6.97)	3.16	0.09	1.65		0.21
Stage REM (%)	24.04 (5.31)	27.00 (4.51)	25.84 (6.02)	0.50	0.49	1.15		0.29
REM density	7.80 (2.66)	5.50 (3.10)	6.40 (3.90)	0.60	0.45	2.45		0.13

Abbreviations: REM, rapid eye movement; SOL, sleep onset latency; TST, total sleep time; WASO, wake after sleep onset. ^a^Log-transformed values were used for the mixed model analyses.

### Power spectral density analyses

As an initial analysis, we compared the total power spectral density in NREM, N3 and REM stages between the first and second night, but no differences were found (all P-values≥0.88). In addition, total power in any stage did not differ between the intervention and control conditions (all P-values ≥ 0.20).

We proceeded with a linear mixed-effects model to examine differences in PSD values in separate frequency bands between the intervention and control conditions. Analyses were run for mean frontal and central powers in five frequency bands (delta: 0.5–3 Hz, theta: 4–7 Hz, alpha: 8–12.5 Hz, beta1: 16–24 Hz, beta2: 25–35 Hz) and separately for N3, NREM, and REM sleep (during the entire night, and for the first sleep cycle).

For the entire night analyses, music listening was significantly associated with PSD in N3 sleep frontal beta1 band (F = 7.50, p = 0.014): beta1 power spectral density was higher in the music listening condition than in the control condition (Estimate = 70.09 vs. 69.77, respectively). Central delta power in N3 was higher in the slow breathing compared to the control condition (F = 5.18, p = 0.036), such that an increased power spectral density was perceived in the slow breathing condition (Estimate = 100.22 vs. 99.51, respectively; Table S1, Supplemental Digital Content).

For the first sleep cycle, music listening condition was not associated with the power spectral density values (Table S2). Slow breathing condition was significantly associated with PSD in frontal (F = 4.64, P < 0.050) and central beta1 (F = 4.64, P < 0.050) band and in central beta2 (F = 5.51, P = 0.03) band during REM sleep, such that the power estimates were higher in the slow breathing condition. (Estimates = 73.33 vs. 72.69; 72.78 vs. 72.08; 65.92 vs. 65.20, respectively).

### Heart rate variability and PSG measures

As a post-hoc analysis, we calculated Pearson correlations between the ANS reactivity to the intervention and PSG variables separately in the music listening and slow breathing conditions.

**Figure 4:**
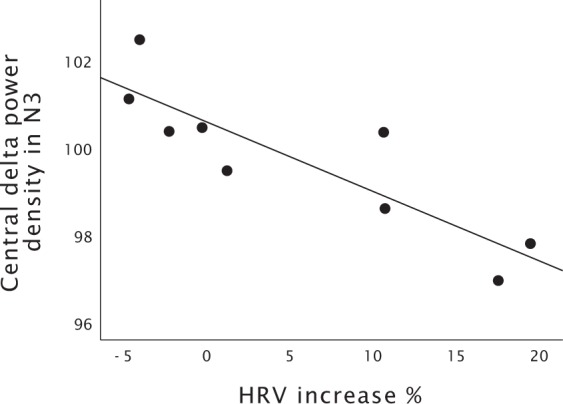
The scatterplot between HRV increase % and central delta power density in N3 sleep.

We found a significant negative correlation in the slow breathing condition between ANS reactivity to the intervention and N3 central delta power spectral density (r = −0.87, P = 0.002, Fig. 4) during the entire night. For the first sleep cycle, none of the correlations were significant. In the music listening condition, no significant correlations between the HRV and PSG variables were found.

## Discussion

The aim of the current pilot trial study was to examine the effects of presleep slow breathing and music listening on PSG-measured sleep structure and sleep power spectrum. The effects of the two conditions were compared to a control situation with no intervention. Both interventions produced an immediate ANS response, with decreasing heart rate and increasing HRV. The increase in HRV continued also at least for 30 minutes after the intervention. Therefore, a temporary increase of parasympathetic activation (LF/HF ratio) was only observed in the slow breathing condition, returning to the baseline after the breathing exercise.

Contrary to the initial hypotheses, we found no effects of the interventions on the length of the sleep latency. The percentage of N2 sleep was decreased, and a non-significant trend was observed for an increased N3 percentage in response, but only in the music listening condition. We also found significant effects of the interventions on EEG power density, such that slow breathing increased delta power over the entire night.

The current study found no effect in PSG-based sleep structure following a presleep slow breathing exercise. Our finding seems to be contradictory to those of two previous studies focusing only on basic sleep parameters. In the first (N = 10, healthy) slow breathing before bedtime resulted in an improved score on an objective sleep disturbance scale^11^. In the second (N = 16, insomniacs), slow breathing at bedtime was followed by a reduced number of awakenings and sleep stage transitions^12^. However, the intervention in the study by Ebben *et al*.^11^ was performed at each participants’ so-called resonance frequency^26^, whereas our study used a pre-defined breathing frequency, which might account for the seemingly conflicting findings. Moreover, in addition to the slow breathing, the intervention in the study by de Zambotti *et al*.^12^ also included visual and auditory stimuli, the latter of which was classical music. Thus, it cannot be ascertained whether the observed effects in their study are attributable to slow breathing, music listening, visual cues, or their combined effects. In one study^10^, presleep slow breathing did not lead to changes in any PSG variables in a group of good sleepers, whereas in a group of self-reported insomniacs, the slow breathing exercise led to improved PSG-measured sleep quality.

Music listening was associated with a lower percentage of stage N2 sleep and a trend towards a higher percentage of stage N3 sleep as compared to control condition. The finding that music listening decreases stage N2 sleep is comparable to results from two previous studies^15,18^. Furthermore, our finding that music listening might increase the percentage of stage N3 sleep is similar to that of Chen *et al*.,^18^ who found that music listening was associated with increased SWS in people with long sleep latency. In another study, increase of SWS was only found among highly suggestible individuals^19^.

Contrary to the observations of our study, one study^15^ showed that listening to music for 45 minutes resulted in increased REM sleep, whereas our study found no such effect. Additionally, a study by Lazic and Ogilvie^17^ reported no effect of music on PSG-measured sleep.

The inconsistencies across previous findings may be attributable several factors. Study samples differ in their participant characteristics, one study excluding those with sleep disturbances^18^, others including only those with self-rated insomnia symptoms^12,15^, and still another including both good and poor sleepers^17^. In some previous studies^12,15,17^, participants started listening to music at bedtime, while trying to fall asleep, whereas in our study, subjects completed the music intervention before going to bed. Additionally, all previous studies^12,15,17,18^ have been conducted in sleep laboratories, or in napping conditions, whereas the present study used more ecologically valid in-home PSG. The inconsistencies observed between our study and previous studies might be partly due to this difference in the measurement location^27,28^. Finally, even though previous studies, as well as the present study, all used some form of sedative, relaxing, or sleep-promoting music, the different effects of specific music pieces cannot be ruled out.

Our study was the first to show that slow breathing associated with increased central delta power during the entire night, indicating that deeper sleep may be obtained with a slow breathing exercise. The effect was not attributable to the first sleep cycle, and interestingly, the higher the ANS response to the intervention was, the smaller the effect was. Further, frontal beta1 power density in N3 sleep during the entire night was higher following music listening. While studies on insomnia suggest that increased beta1 power is a marker of cortical hyperarousal^29,30^, our study cannot conclude that the found effect would be sleep disturbing with the absence of other poor sleep quality markers. Instead, Yordanova *et al*. observed higher beta power to reflect memory reprocessing and, assumedly, synchronized network activity, which provoked us to speculate if pre-sleep music listening would facilitate such activity^31^. Similar increases in beta activity were observed in the first sleep cycle REM sleep following slow breathing.

We found no effect on delta power in the music listening condition, which appears to contradict a previous study^17^, where the mean delta power at temporal locations showed elevated trends in the music listening than in the control conditions. Methodological differences are evident. In the study of Lazic and Ogilvie^17^ stimuli were continued for five minutes after the participants fell asleep, and EEG was analyzed in brief periods at sleep onset and at the start of slow-wave sleep. However, restricting the power density analyses to the first sleep cycle did not change our null result. More research combining different relaxation interventions and sleep EEG is warranted.

### Strengths and limitations

The strengths of the current study include a randomized, controlled design and the use of objective assessment of sleep quality by PSG. PSG recording took place at participants’ homes, which allows for the collection of data in participants’ usual sleeping environment. Participants of our study were encouraged to follow their usual sleep times and routines, increasing the ecological validity of the study. However, lack of information on the prior nights’ sleep may bias our findings.

Our study used a relatively small sample size, detecting only large effect sizes. The sample consisted mostly of university students, so it is not known whether the results can be generalized to other age groups or clinical populations. Additionally, it was not possible to blind subjects to their experimental condition, which might have introduced bias. Moreover, as this is a small study and the hypotheses were not independent of each other, we did not correct for multiple testing. The significance levels must be thus interpreted with caution.

The present study, as well as several previous studies^10,11,17,18^, have studied the effects of only one intervention night on sleep. Previous studies using subjective assessment of sleep have shown that music listening^32,33^ and slow breathing^34^ can improve subjective sleep quality if practiced over several weeks. Moreover, it has been shown that music can have a cumulative effect on sleep quality, in that the effects of music increase over time^32,33,35,36^. Slow breathing, on the other hand, can be uncomfortable when practiced for the first time^7^, thus limiting its possible relaxing and stress-reducing effects. Therefore, further studies should use objective measures of sleep to evaluate the effects of music and breathing interventions that span over longer periods of time, such as days or weeks.

## Conclusions

There is an urgent need for new evidence-based and non-pharmacological methods to improve sleep quality. The present study provides evidence that, in healthy young adults with no diagnosed sleep disorders, slow breathing exercise prior to sleep may increase delta power in SWS over the entire night, and beta1 power during the first REM episode. Presleep music listening improved sleep by decreasing stage N2 and increasing stage N3 sleep, although the latter association was not statistically significant. The current study also provides tentative evidence that music listening may increase frontal beta1 power in SWS over the entire night.

While this study was explorative in nature our results indicated that presleep relaxation influences several aspects of sleep. We suggest that subsequent studies should apply more prolonged intervention periods, as slow breathing may require practicing before being beneficial. Overall, the findings should be interpreted with caution, and replications with larger sample sizes and more diverse subject populations are needed.

## Supplementary information

Supplement tables.
