# Supplementary material for: The Effects of Presleep Slow Breathing and Music Listening on Polysomnographic Sleep Measures – a pilot trial
Source: Sci Rep. 2020 May 4;10:7427. doi: 10.1038/s41598-020-64218-7 (PMC7198497; doi:10.1038/s41598-020-64218-7)
Supplement: Supplementary file 1 — Supplement tables. [file 41598_2020_64218_MOESM1_ESM.docx]

**The Effects of Presleep Slow Breathing and Music Listening on Polysomnographic Sleep Measures – a pilot trial**

Liisa Kuula, Risto Halonen, Kristiina Kajanto, Jari Lipsanen, Tommi Makkonen, Miina Peltonen, Anu-Katriina Pesonen

Table S1

*Results of the Mixed-Effects Model Analyses for Mean PSD values in both intervention conditions during the entire night.*

|  | Music listening | | Slow breathing | |
| --- | --- | --- | --- | --- |
|  | F | p | F | p |
| N3 |  |  |  |  |
| Delta (frontal) | 0.82 | .38 | 1.82 | .20 |
| Delta (central) | 1.41 | .25 | 5.18 | .036 |
| Theta (frontal) | 0.79 | .39 | 0.02 | .90 |
| Theta (central) | 1.84 | .19 | 0.96 | .34 |
| Alpha (frontal) | <0.01 | .96 | <0.01 | .99 |
| Alpha (central) | 0.44 | .51 | 0.60 | .45 |
| Beta1 (frontal) | 7.45 | .014 | 0.41 | .53 |
| Beta1 (central) | 2.83 | .11 | 0.06 | .81 |
| Beta2 (frontal) | 2.36 | .14 | 0.81 | .39 |
| Beta2 (central) | 3.28 | .09 | 0.23 | .64 |
| NREM |  |  |  |  |
| Delta (frontal) | 0.44 | .52 | 1.48 | .24 |
| Delta (central) | 0.39 | .54 | 2.15 | .16 |
| Theta (frontal) | 0.27 | .61 | 0.34 | .57 |
| Theta (central) | 0.77 | .39 | 0.93 | .35 |
| Alpha (frontal) | 0.82 | .38 | 0.88 | .36 |
| Alpha (central) | 0.61 | .45 | 0.53 | .48 |
| Beta1 (frontal) | 0.26 | .62 | 0.18 | .68 |
| Beta1 (central) | 0.39 | .54 | 0.32 | .58 |
| Beta2 (frontal) | 1.31 | .27 | 0.29 | .60 |
| Beta2 (central) | 1.48 | .24 | 0.21 | .65 |
| REM |  |  |  |  |
| Delta (frontal) | 1.55 | .23 | 0.26 | .62 |
| Delta (central) | 0.27 | .61 | 0.06 | .82 |
| Theta (frontal) | 0.34 | .57 | 0.41 | .53 |
| Theta (central) | 0.45 | .51 | 1.67 | .22 |
| Alpha (frontal) | 0.22 | .65 | 0.70 | .41 |
| Alpha (central) | 0.07 | .80 | 0.58 | .46 |
| Beta1 (frontal) | 0.08 | .78 | 0.60 | .45 |
| Beta1 (central) | 0.02 | .88 | 1.57 | .23 |
| Beta2 (frontal) | 0.06 | .81 | <0.01 | .99 |
| Beta2 (central) | 0.00 | 1.00 | <0.01 | .98 |

Table S2

*Results of the Mixed-Effects Model Analyses for Mean PSD values in both intervention conditions during the 1^st^ sleep cycle.*

|  | Music listening | | Slow breathing | |
| --- | --- | --- | --- | --- |
|  | F | p | F | p |
| N3 |  |  |  |  |
| Delta (frontal) | <0.01 | .97 | .29 | .60 |
| Delta (central) | 0.82 | .38 | <0.01 | .96 |
| Theta (frontal) | <0.01 | .93 | 0.02 | .90 |
| Theta (central) | 0.31 | .59 | 0.21 | .65 |
| Alpha (frontal) | 0.21 | .66 | 0.89 | .36 |
| Alpha (central) | 2.75 | .12 | 2.83 | .11 |
| Beta1 (frontal) | 2.19 | .16 | 0.87 | .36 |
| Beta1 (central) | 2.12 | .16 | 1.48 | .24 |
| Beta2 (frontal) | 3.77 | .07 | 0.46 | .51 |
| Beta2 (central) | 3.74 | .07 | 0.50 | .49 |
| NREM |  |  |  |  |
| Delta (frontal) | 0.02 | .90 | 0.37 | .55 |
| Delta (central) | 0.01 | .92 | 0.10 | .75 |
| Theta (frontal) | <0.01 | .99 | 2.15 | .16 |
| Theta (central) | 0.06 | .81 | 0.82 | .38 |
| Alpha (frontal) | <0.01 | .96 | 0.12 | .73 |
| Alpha (central) | 0.08 | .79 | 0.15 | .70 |
| Beta1 (frontal) | 0.09 | .76 | 0.64 | .44 |
| Beta1 (central) | 0.07 | .79 | 1.17 | .29 |
| Beta2 (frontal) | 0.98 | .33 | 0.02 | .89 |
| Beta2 (central) | 0.88 | .36 | 0.04 | .84 |
| REM |  |  |  |  |
| Delta (frontal) | 2.93 | .11 | 1.19 | .29 |
| Delta (central) | 2.00 | .18 | .045 | .51 |
| Theta (frontal) | 1.80 | .20 | 1.72 | .21 |
| Theta (central) | 0.05 | .82 | 0.14 | .72 |
| Alpha (frontal) | 1.40 | .25 | 1.16 | .30 |
| Alpha (central) | 1.46 | .25 | 0.73 | .41 |
| Beta1 (frontal) | 2.98 | .11 | 4.64 | .050 |
| Beta1 (central) | 3.61 | .08 | 6.11 | .027 |
| Beta2 (frontal) | 0.88 | .36 | 3.38 | .09 |
| Beta2 (central) | 1.55 | .23 | 5.51 | .033 |
